# Supplementary material for: Analysis of sensitive information leakage in functional genomics signal profiles through genomic deletions
Source: Nat Commun. 2018 Jun 22;9:2453. doi: 10.1038/s41467-018-04875-5 (PMC6015012; doi:10.1038/s41467-018-04875-5)
Supplement: Supplementary file 1 — Supplementary Information [file 41467_2018_4875_MOESM1_ESM.pdf]

Supplementary Information for “Analysis of Sensitive Information Leakage in Functional Genomics Signal Profiles through Genomic Deletions”, Harmanci *et al.*

Supplementary Note 1: Example Linking Attack: Netflix Prize Competition

A well-known example of a linking attack is the Netflix Prize Competition(Narayanan & Shmatikov, 2008) (Supplementary Fig. 1a,b). In this competition, the movie rental company Netflix released an anonymized dataset that was to be used for training new automated movie rating algorithms. Although the dataset seemed safe to share at the time, the training dataset was linked to the seemingly independent database of the Internet Movie Database. The linking revealed movie preferences and identities of many Netflix users. We believe that similar scenarios will be a major route to breaches in individual genomic privacy.

Supplementary Note 2: Details of the Linking Attack Scenario

Figure 1 summarizes the linking attack scenario that we are studying. The attack involves cross-referencing the individuals in a signal profile dataset (denoted by S) against the individuals in a genotype dataset, denoted by G. The signal profile dataset is publicly available and contains a genome-wide signal profile and an anonymized identifier for each individual. The signal profile for an individual represents the measurements of functional activity at each genomic position. In addition, the signal profile dataset contains sensitive information about each individual (e.g., HIV status). We assume that the dataset is generated for research purposes and is publicly released. The genotype dataset, G, contains, for each individual, the genotypes of a panel of structural variants, denoted by p_G. The genotype dataset also contains the identities of the individuals. Thus, G is normally assumed to be protected. We assume that the adversary obtains access to G. This accession could be established by lawful or unlawful means. For example the adversary might have stolen it or might have been legally allowed to access it but violated the terms of data accession. The main objective of the adversary is to link G and S by first predicting the structural variant genotypes from signal profiles in S, and then matching the predicted genotypes to the genotypes in G. For any matching individuals in G and S, the name and sensitive information are revealed to the adversary.

The attack has two steps. The first step is genotyping the deletion variants, which is illustrated in Figure 1a. The adversary has access to a genome-wide signal profile dataset (S) for a sample of individuals. This dataset stores a genome-wide signal profile for each individual, for example containing RNA-Seq or ChIP-Seq data. In the first scenario, we assume that the adversary has access to a reference panel of genomic structural variant loci, which are denoted by p_S. For each individual, the adversary utilizes the signal profile and genotypes the deletions in p_S. After genotyping, the adversary builds a data matrix with the predicted genotypes, which is denoted by $\tilde{G}$. We refer to this scenario as linking based on "genotyping only". The second scenario, also illustrated in Figure 1a, is very similar except that the adversary does not have access to, but discovers the panel of structural variants from the signal profiles. The adversary then uses the signal profiles to genotype the SVs in this *de novo*-discovered SV panel. We refer to this scenario as linking based on "joint discovery and genotyping". After genotyping, the genotyped SV matrix ($\tilde{G}$) includes, for each individual, the predicted SV genotypes and the sensitive information (e.g., HIV status). $\tilde{G}$ can also be thought of as a noisy genotype matrix, since the genotype predictions may contain errors.

The second step of the linking attack is cross-referencing the individuals in the genotyped SVs ($\tilde{G}$) and the individuals in the genotype dataset, G, illustrated in Figure 1b. The SV genotype dataset G is assumed to contain identifying information about the individuals. Thus, we assume that this dataset was previously

protected but was either leaked or stolen (e.g., variants from a glass). The adversary first compares the genotyped SV panel (p_S) to the SV panel of the genotype dataset, which is denoted by p_G. After matching the SVs in the two panels, the adversary compares the genotypes of the matching SVs in the two panels. The adversary uses this comparison to cross-reference the individuals in two datasets and find the individuals that best match each other with respect to genotype match distance (i.e., links individuals in two datasets). The results are used to link the individuals in genotype dataset to those in the signal profile dataset and the sensitive information, e.g., HIV status of individuals in the genotype dataset are revealed to the adversary (the matched columns in the final linked matrix).

Supplementary Note 3: Information Content and Predictability of SV Genotypes

In order to assess the correct predictability of SV genotypes, we propose using genome-wide predictability of SV genotypes, denoted by π_{GW}, from signal profiles. Predictability measures how accurately an SV genotype can be estimated given the signal profile. The predictability of the genotype of a structural variant is the conditional probability of the variant genotype given the signal profile. By this definition, predictability only depends on the genomic signal levels of an individual and how well they can be used to predict genotypes. In principle, genome-wide predictability is computed for each individual independently. Therefore, the genome-wide predictability of a variant from a signal profile is independent from the population frequency of the variant. This is discussed more below.

Other than predictability, an important measure in linking attacks is the information content each SV genotype supplies. We utilized a previously proposed metric termed individual characterizing information (ICI) to quantify the information content of each SV(Harmanci & Gerstein, 2016). For a given variant genotype, ICI measures how much information it supplies for pinpointing an individual in a population. This measure gives higher weight to genotypes that have low population frequency. As the genome-wide predictability is independent of the population frequency of the variants, the adversary can utilize genome-wide prediction approaches and predict rare variant genotypes to gain high ICI and characterize individuals accurately. This is an important distinction between the genome-wide predictability that is presented here and the population-wide predictability that is presented in an earlier study. The population-wide predictability depends substantially on the population frequency of the genotypes and it is low for genotypes with low frequency (i.e., high ICI). On the other hand, genome-wide predictability can be very high even for variants that are very low in frequency. This makes it possible for an adversary to use genome-wide prediction to uncover variant genotypes that are highly identifying.

Supplementary Note 4: Breadth and Depth Coverages of Signals

The first is breadth of coverage, which measures how well the genome is covered by signal profiles. The second is depth of coverage, which measures how deep the sequencing is performed. DNA-sequencing read depth signal(Abyzov, Urban, Snyder, & Gerstein, 2011; Handsaker, Korn, Nemesh, & McCarroll, 2011) is very suitable for the detection of deletions because it uniformly cover the genome (high-breadth coverage) in a deep manner (high-depth coverage). On the other hand, the detection of genomic deletions from functional genomics datasets is not as straightforward. The main reason for this is the dynamic and non-uniform nature of the signal profiles of functional genomics experiments. For example, RNA-Seq(Wang, Gerstein, & Snyder, 2009) signal profiles are concentrated mainly on the exonic regions. RNA-Seq signal profiles generally have high-depth but low-breadth coverage. This makes RNA-Seq signal

profiles very suitable for detecting small deletions in exonic regions. We show that a large number of small deletions can be detected using RNA-Seq signal profiles. Chromatin immunoprecipitation sequencing (ChIP-Seq)(Pepke, Wold, & Mortazavi, 2009) signal profiles for diffuse histone modifications (such as H3K9me3) generally have high breadth but low depth of coverage. These modifications are suitable for detecting large deletions. In addition, these experiments are generally done in combination. This is important because although each experiment assays a different type of genome-wide activity, pooling the signal profiles increases both the depth and breadth of coverage and can bring enough power to an adversary for genotyping large deletions and performing successful linking attacks.

Supplementary Note 5: Linking Attacks using RNA-Seq Signal Profiles

We first focused on the predictability of small deletions using RNA-Seq signal profiles. Figure 1d illustrates a hypothetical example of how small deletions in RNA-Seq signal profiles can be detected as small and sudden dips in the signal. As an example showing the relevance of small deletions in RNA-Seq signal profiles, we include a screenshot of signal profiles around a small deletion for six individuals in the GTEx Project (Supplementary Fig. 3). The two base pair deletion, rs34043625, can be easily detected for three of the individuals shown. An important aspect of the effect of small deletions on the signal profile is the extent to which they affect the total expression of a gene. It is clear from Supplementary Figure 3 that the total signal in the small dips in the RNA-Seq signal is much smaller than the perturbations caused by other genetic factors like eQTLs and splicing QTLs. In general, an eQTL is associated with a global change in the total signal on a RNA-Seq signal profile of a gene. However, a small deletion affects a localized position on the RNA-Seq signal profile with a relatively smaller effect on the total expression of the gene, assuming the small deletion is not an eQTL.

Supplementary Note 6: Challenges Around Open Sharing Functional Genomics Datasets

Another important factor is the desire to share and study RNA-Seq datasets to help find cures for various diseases. Because of this, there is great incentive to find ways to share functional genomics data without privacy protections. The large scale protections do not allow researchers to share results openly on the web. Consequently, many consortia, such as the Genotype-Tissue Expression (GTEx) Project, aim to share RNA-Seq information to the maximum extent. Although the raw reads cannot be shared, there is a general belief that other aggregated data computed using raw reads, such as signal profiles and gene-level quantifications, can be shared. Signal profiles simply reflect the overall depth of coverage of the RNA-Seq reads at any given position on the genome (Supplementary Fig. 2a). Many consortia openly share RNA-Seq signal profiles because they ostensibly do not contain variant information. In this study, we focus on leakage from signal profiles. Another commonly shared aggregated data are gene-level quantifications, which are essentially averages of the signal profile over exons. Although overall aggregation and averaging reduces information, private information leakage also decreases. However, private information leakage still occurs from gene expression quantifications through the association of expression levels with variants called expression quantitative trait loci (eQTLs). Although we do not tackle this in the current study, it has been explored elsewhere(Harmanci & Gerstein, 2016; Schadt, Woo, & Hao, 2012).

Fig S1a

What is a linking attack?

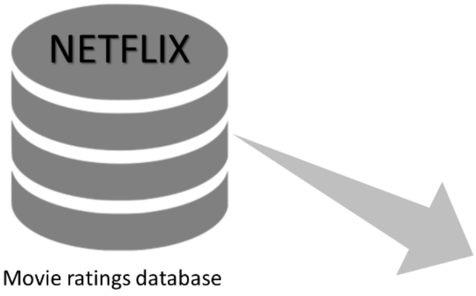

Anonymized Netflix Prize Training Dataset made available to contestants

100 million ratings
500,000 users

200 movie ratings/user
5,000 users/movie rating

User (ID)	Movie (ID)	Date of Rating	Rating [1,2,3,4,5]
NTFLX-0	NTFLX-19	10/12/2008	1
NTFLX-1	NTFLX-116	4/23/2009	3
NTFLX-2	NTFLX-92	5/27/2010	2
NTFLX-1	NTFLX-666	6/6/2016	5
…	…	…	…
…	…	…	…

Fig S1b

Linking Attacks: Case of Netflix Prize

NETFLIX ⟷ IMDb

User (ID)	Movie (ID)	Date of Grade	Grade [1,2,3,4,5]
NTFLX-0	NTFLX-19	10/12/2008	1
NTFLX-1	NTFLX-116	4/23/2009	3
NTFLX-2	NTFLX-92	5/27/2010	2
NTFLX-1	NTFLX-666	6/6/2016	5
…	…	…	…
…	…	…	…

Names available for many users!

User (ID)	Movie (ID)	Date of Grade	Grade [0-10]
IMDB-0	IMDB-173	4/20/2009	5
IMDB-1	IMDB-18	10/18/2008	0
IMDB-2	IMDB-341	5/27/2010	-
…	…	…	…
…	…	…	…
…	…	…	…

Supplementary Figure 1: Illustration of the Netflix Prize competition and linking to IMDb. a) Netflix released an anonymized training dataset that contained the movie identifiers, ratings, dates of ratings, and anonymized user identifiers. This dataset contained more than 100 million ratings for 500,000 users where each user had rated an average of 200 movies and each movie was rated on average by 5,000 users. b) The training dataset was linked to IMDb's database. The linking is based on matching the movie rating, the date of rating and other features in the databases. For the individuals whose names can be found in the IMDb database, the movie ratings are made public.

Fig S2a

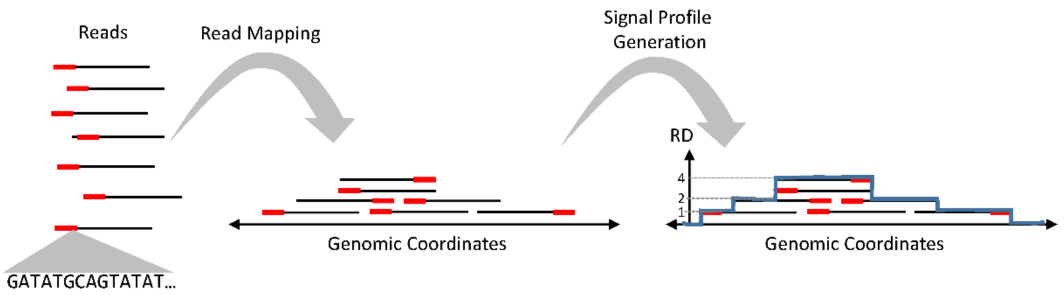

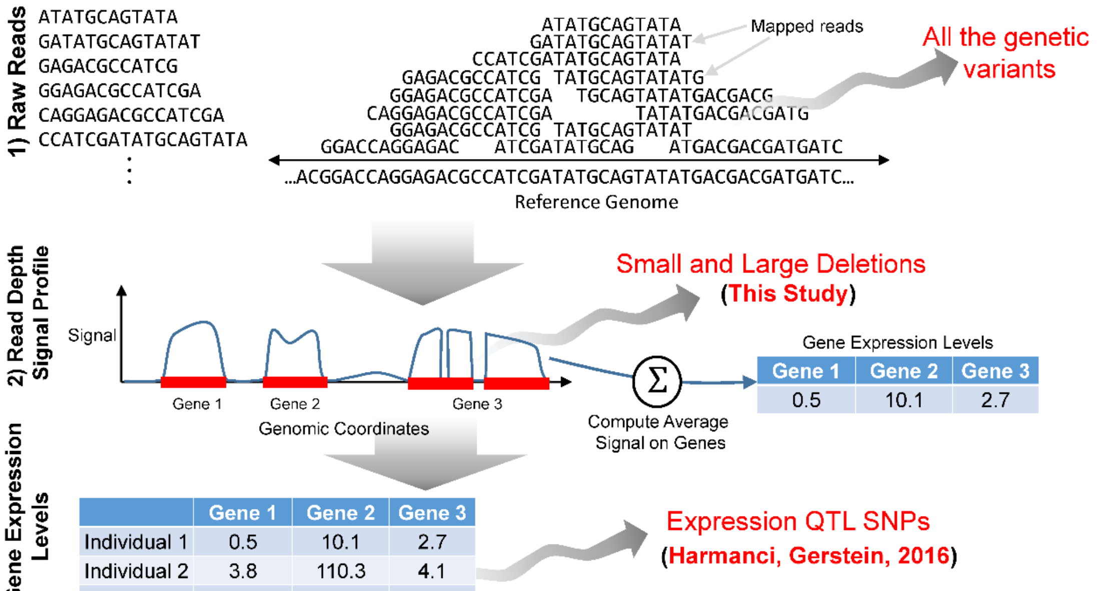

Fig S2b

Supplementary Figure 2: Illustration of different representations of sequencing data. a) Figure shows creation of read depth signal profiles. The reads are shown with black lines. The red dashes indicate the 5' ends of reads. The read mapping process maps the reads on reference genome. The reads have either forward or reverse orientation. The read depth signal profile (shown in the rightmost part) is constructed by counting the number of reads that overlap with each position on the genome. The y-axis on the rightmost figure shows the read depth for the mapped reads. The profile is shown with the heavy grey line on the mapped reads. b) Illustration of the sources of leakage from different representations of sequencing data. The raw reads (shown on top of the stack) leak all the genetic variants. The read depth signal profiles (shown in the middle section) leak the genomic deletions. This manuscript deals with this leakage. The signal profile is integrated over genes to compute the gene expression levels, as illustrated with the table. The gene expression levels (shown at the bottom of the stack) leak the eQTL variant genotypes. In this scenario, the table of gene expression levels are used to genotype eQTLs variants. This leakage was previously studied.

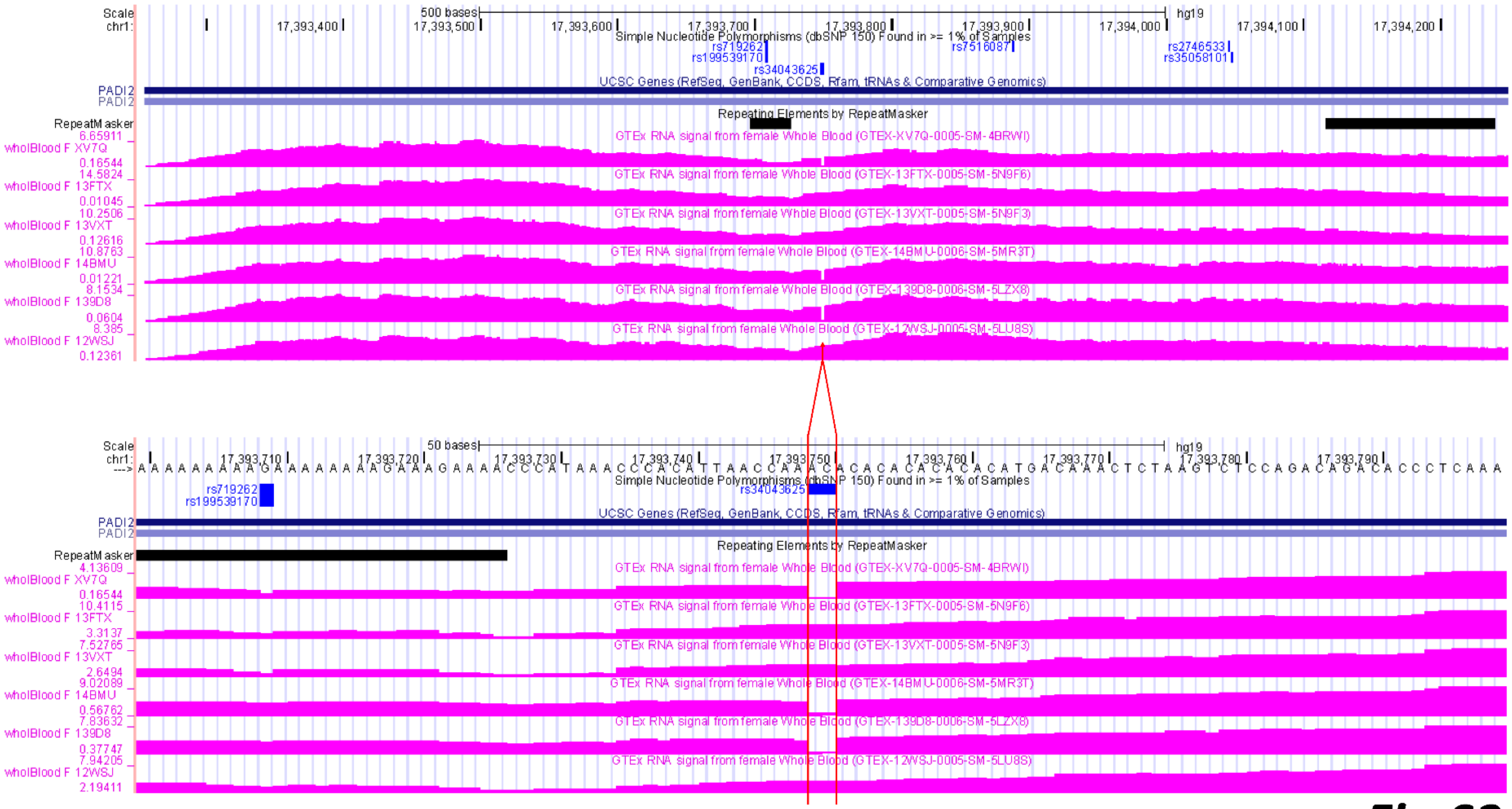

Fig S3

Supplementary Figure 3: A screenshot of the region surrounding the two base pair indel rs24043625 in GTEx RNA-Seq signal profile hub on UCSC Genome Browser. The figure shows the profiles for 6 individuals. The top figure shows 6 kb region around the deletion. The bottom figure shows a zoomed view around 100 base pairs of the deletion. The dip in the RNA-Seq signal that is caused by the deletion is visible by eye in three individuals, XV7Q, 14BMU, and 139D8. Other individuals shown in the figure do not have this deletion. It is also worth noting that these signal profiles are publicly available for download from the UCSC Genome Browser.

Fig S4 Population-wide predictability of 1kG SVs

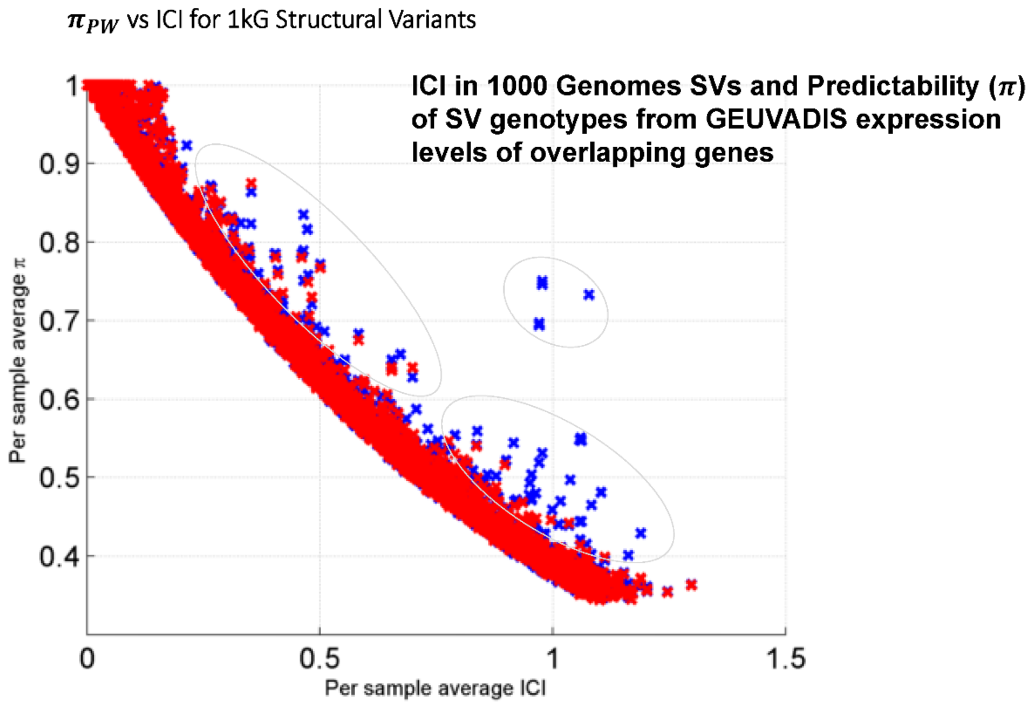

Supplementary Figure 4: A scatter plot of population-wide predictability versus ICI leakage of the SV genotypes when gene expressions are used to genotype SVs. Each dot represents a 1000 Genomes SV and the population-wide predictability represents how correctly predictable the SV genotypes are given the gene expression levels. Gene expression levels were obtained from the GEUVADIS dataset. The ellipses point to the small number SVs that have high predictability and high ICI leakage.

Fig S5 Features for genotyping deletions

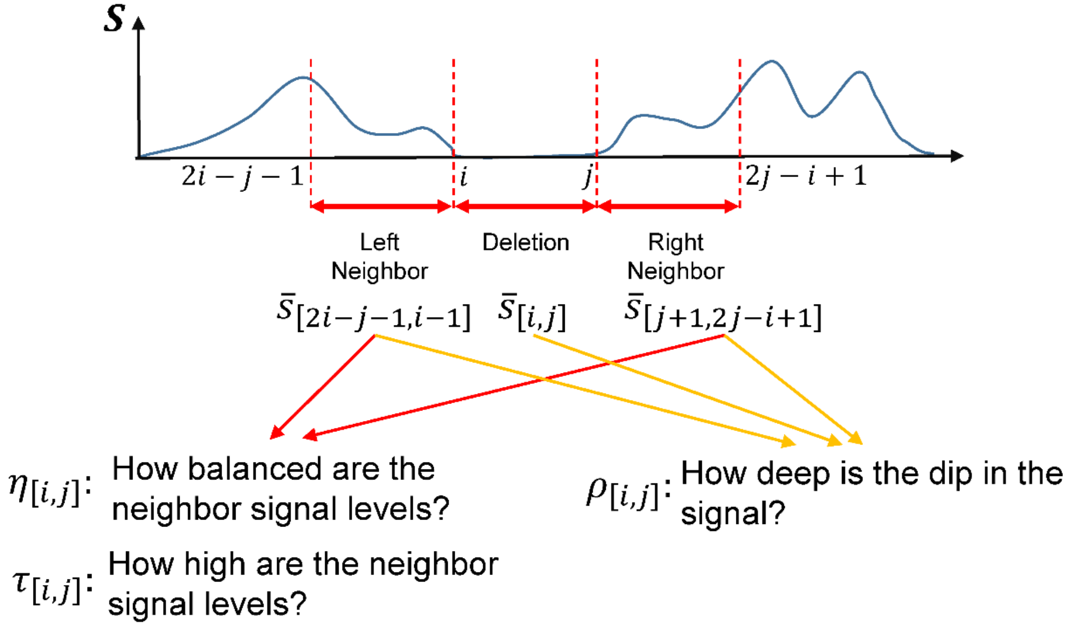

Supplementary Figure 5: Feature sets that are used to genotype and discover deletions. A candidate deletion is located between the i and j indices. The attacker uses the signal profiles within the deletion region and the left and right neighboring regions ($[2i-j-1, i-1]$ and $[j+1,\ 2j-i+1]$, respectively) to compute the features. $\rho_{[i,j]}$ represents the deepness of the dip in the signal profile along the deletion. $\eta_{[i,j]}$ represents how balanced the signal levels in the neighboring regions are. $\tau_{[i,j]}$ represents how high the signal levels are in the neighboring regions.

Fig S6

Genotype prediction accuracy

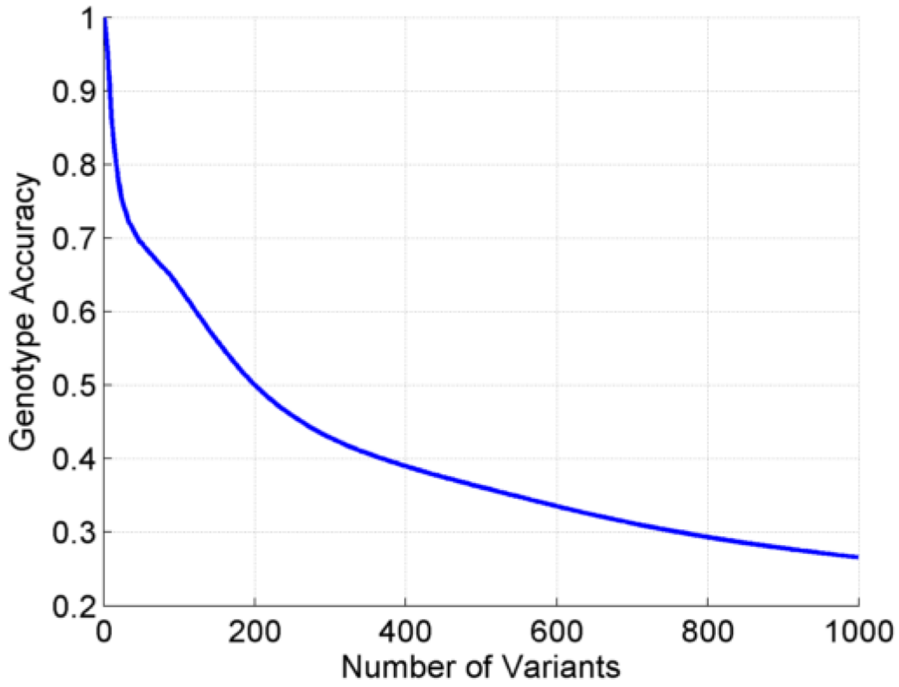

Supplementary Figure 6: Accuracy of genotype predictions that are used in instantiating the linking attacks. The x-axis shows the number of variants used and the y-axis shows the genotype accuracy. The GEUVADIS signal profiles are used with a known panel of 1000 Genomes small indels.

REFERENCES

Abyzov, A., Urban, A. E., Snyder, M., & Gerstein, M. (2011). CNVnator: An approach to discover, genotype, and characterize typical and atypical CNVs from family and population genome sequencing. *Genome Research*, *21*(6), 974–984. https://doi.org/10.1101/gr.114876.110

Handsaker, R. E., Korn, J. M., Nemesh, J., & McCarroll, S. A. (2011). Discovery and genotyping of genome structural polymorphism by sequencing on a population scale. *Nature Genetics*, *43*(3), 269–276. https://doi.org/10.1038/ng.768

Harmanci, A., & Gerstein, M. (2016). Quantification of private information leakage from phenotype-genotype data: linking attacks. *Nature Methods*, *13*(3), 251–256. https://doi.org/10.1038/nmeth.3746

Narayanan, A., & Shmatikov, V. (2008). Robust de-anonymization of large sparse datasets. In *Proceedings - IEEE Symposium on Security and Privacy* (pp. 111–125). https://doi.org/10.1109/SP.2008.33

Pepke, S., Wold, B., & Mortazavi, A. (2009). Computation for ChIP-seq and RNA-seq studies. *Nature Methods*, *6*, S22–S32. https://doi.org/10.1038/nmeth.1371

Schadt, E. E., Woo, S., & Hao, K. (2012). Bayesian method to predict individual SNP genotypes from gene expression data. *Nature Genetics*. https://doi.org/10.1038/ng.2248

Wang, Z., Gerstein, M., & Snyder, M. (2009). RNA-Seq: a revolutionary tool for transcriptomics. *Nature Reviews. Genetics*, *10*(1), 57–63.
